# A stochastic model describing transport of PSD-95 molecules in spiny dendrites provides the basis for synaptic plasticity

**DOI:** 10.1186/1471-2202-12-S1-P96

**Published:** 2011-07-18

**Authors:** Dmitry Tsigankov, Stephan Eule

**Affiliations:** 1 Max-Planck Institute for Dynamics and Self-Organization, Goettingen, 37073, Germany; 2 Bernstein Center for Computational Neuroscience, Goettingen, 37073, Germany

## 

Molecules in neurons are in a state far from equilibrium. Therefore their transport properties are strongly affected by fluctuations. We present a model of stochastic molecular transport in neurons which have their synapses located in the spines of a dendrite. In this model we assume that the molecules perform a random walk between the spines that trap the walkers. If the molecules are assumed to interact with each other inside the spines, the trapping time in each spine depends on the number of molecules in the respective trap. The corresponding mathematical problem has non-trivial solutions even in the absence of external disorder due to self-organization phenomena. We obtain the stationary distributions of the number of walkers in the traps for different kinds of on-site interactions between the walkers and furthermore analyze how birth and death processes of the random walkers affect these distributions.

We apply this model to describe the dynamics of the PSD-95 proteins in spiny dendrites. PSD-95 is the most abundant molecule in the post-synaptic density (PSD) located in the spines. It is observed that these molecules have high turnover rates and that neighboring spines are constantly exchanging individual molecules. Thus we predict the distribution of PSD-95 cluster sizes that determine the size of the synapse and thus the synaptic strength. Finally, we show that in the model non-equilibrium inter-spine dynamics of PSD-95 molecules can provide the basis for locally controlled synaptic plasticity through activity-dependent ubiquitinization of PSD-95 molecules.

